# Improving measures of context in process evaluations: development and use of the Context Tracker tool

**DOI:** 10.1186/s13063-024-08623-7

**Published:** 2024-11-18

**Authors:** Joanna Busza, Fortunate Machingura, Cedomir Vuckovic

**Affiliations:** 1 Centre for Evaluation, London School of Hygiene and Tropical Medicine, Keppel Street, London, WC1E 7HT UK; 2 Centre for Sexual Health and HIV/AIDS Research (CeSHHAR) Zimbabwe, 4 Bath Road, Harare, Zimbabwe; 3 UNICEF Innocenti – Global Office of Research and Foresight, Via Degli Alfani, 58, Florence, 50121 Italy

**Keywords:** Method, Process evaluation, Context, Marginalised populations

## Abstract

**Background:**

Process evaluations are increasingly integrated into randomised controlled trials (RCTs) of complex interventions to document their delivery and interactions with local systems and dynamics, helping understand observed health outcomes. Yet process evaluations often struggle to assess relevant contextual determinants, leaving much of the important role of “context” in shaping an intervention’s mechanisms opaque in many studies. A lack of easily adapted data collection methods to help define and operationalise indicators of context likely contributes to this.

**Methods:**

We present a method to help structure measures of context in process evaluations and describe its use in two very different settings. The “Context Tracker” is an innovative tool for use within trials and quasi-experiments to more systematically capture and understand key dimensions of context. It was developed in Zimbabwe as part of a cluster randomised controlled trial and then adapted for a quasi-experimental evaluation in the UK. Both studies provided harm reduction and health services for marginalised and hard-to-reach populations.

**Results:**

We developed the Context Tracker to be both standardised (i.e. formatted and applied in the same way across study sites) and flexible enough to allow unique features to be explored in greater detail. Drawing on the Context and Implementation of Complex Interventions (CICI) and Risk Environments frameworks, we mapped 5 domains across micro, meso and macro levels in a simple table and used existing evidence and experience to predict factors likely to affect delivery of and participation in intervention components. We tracked these over time across study sites using routine programme statistics, observation and qualitative methods. The Context Tracker enables identification and comparison of facilitators and barriers to implementation, variations in engagement with interventions, and how mechanisms of action are (or are not) triggered in different settings.

**Conclusions:**

The Context Tracker is one example of how evidence-based contextual determinants can be used to guide data collection and analysis within process evaluations. It is relevant in low- and high-income settings and applicable to both qualitative and quantitative analyses. While perhaps most useful to process evaluations of complex interventions targeting marginalised communities, the broader approach would benefit a more general research audience.

## Background

Rigorous evaluation of complex health interventions is necessary to ensure effectiveness of programmes taken up in policy and practice [1]. Comprehensive process evaluations are increasingly integrated into randomised controlled trials (RCT) and quasi-experiments to document the delivery of intervention activities and their interactions with local systems and dynamics, helping to understand observed health outcomes [2, 3]. Process evaluations examine the implementation of health interventions, test their theoretical foundations by tracking whether delivered components lead to hypothesised changes, and explore the means through which they produce outcomes. There are numerous frameworks to guide process evaluation design, all of which include the need to capture “context” as characteristics and attributes of the setting into which interventions are introduced. The widely used Medical Research Council (MRC) Guidance on process evaluations included context as one of its 3 pillars, alongside implementation and mechanisms of action [2]. However, while specific domains for researchers to operationalise were assigned to the other pillars, context was simply defined as “anything external to the intervention that may act as a barrier or facilitator to its implementation, or its effects” [2].

This initial gap has now been filled by a range of conceptual models offering lists and categories of contextual domains and suggestions for how they might interact with one another to moderate both interventions and outcomes [4, 5]. The revised MRC Guidance acknowledges that context can change during implementation, both as a result of the intervention and for externally driven reasons, e.g. an unrelated policy change [6]. Checklists for reporting design and evaluation of health interventions often equate context with characteristics of the intervention setting [7, 8], which can be further classified, e.g. into geographical, epidemiological, socio-cultural, socio-economic, ethical, legal and political attributes [9]. Realist evaluation approaches highlight the importance of context by defining interventions as new resources and opportunities that, when introduced into a specific context, trigger specific mechanisms of change [10]. By asking “what works, for whom, and in what circumstances?”, realist evaluators blur the separation between an intervention and where it is delivered [11]. Nonetheless, recent reviews have found “context” remains inadequately conceptualised and measured in process and outcome evaluations [12–14]. A lack of easily adapted data collection methods and practical guidance to help define and operationalise indicators of context likely contributes to this and authors have commented on the dearth of tools to apply during process evaluations [5, 15].

What guidance exists for incorporating contextual measures into the design of process evaluations often refers to the importance of having a clear logic model that sets out how an intervention’s components are hypothesised to lead to intended outcomes. Logic models (also referred to as logical frameworks, programme theories or theories of change) specify the pathway from an intervention’s activities to effects on systems, behaviours, determinants of health and, ultimately, health outcomes, thus providing the underlying rationale for the intervention [16, 17]. An explicit narrative or diagrammatic map facilitates empirical testing of each step in the pathway, for which indicators and measures are identified in advance. Assumptions, including about contextual facilitators and barriers to success, can be added although, as highlighted by realist evaluators, often are not [18]. Thus while many process evaluations specify data to be collected to track progress along the change pathway, documenting contextual factors tends to rely on ad hoc analysis of information collected for other purposes, often through observations by the research team or interviews with implementors, and thus is limited to factors they consider noteworthy [19]. It is possible, however, to predict key contextual factors in advance based on existing literature and previous experience, particularly local knowledge of implementing partners and participating communities, while remaining flexible and open to unforeseen events or effects [20]. For example, where an intervention addresses a socially sensitive topic, e.g. sexuality education for adolescents, local parental attitudes, religiosity and community trust in school leadership are likely to be key shapers of both delivery and results [21, 22]. Early consideration of potential influencers in each domain of a chosen conceptual framework can increase the likelihood that the process evaluation will capture data that will contribute findings useful for transferring interventions to new settings or delivering at scale outside of carefully controlled research conditions.

## Methods

In this paper, we present a method to help plan measures of “context” in process evaluations and describe its use in two very different settings. The “Context Tracker” is an innovative tool for use within trials and quasi-experiments to more systematically capture and understand key dimensions of context. It was initially developed in Zimbabwe as part of a cluster randomised controlled trial (cRCT) and then adapted for a quasi-experimental evaluation of a pilot project in the UK. Although the interventions introduced in each study differed considerably, both addressed infectious disease transmission among criminalised and marginalised (“key population”) communities, which share structural vulnerabilities [23–25].

Interventions for marginalised populations can be particularly susceptible to the influence of local context. Health interventions targeting populations with poor relationships with authorities have to navigate contextual barriers related to restrictive legal policy and its enforcement, and consequent suspicion and distrust by community members [26, 27]. For example, drug use and sex work (sale and purchase of sex) are widely stigmatised and criminalised, with individuals involved in these behaviours considered social deviants, vectors of disease and blamed for their disproportionate risk of adverse health outcomes such as blood-borne and sexually transmitted infections, e.g. HIV and hepatitis C [28, 29]. Evaluations of interventions for these communities broadly refer to “context” as legal frameworks and restricted access to health services, but there are highly nuanced differences in how punitive laws, policing strategies and social stigma are locally enacted. These influence facilitators and barriers to implementing activities to reach, engage and provide services and support to marginalised communities, and also shape individuals’ motivation and self-efficacy to interact with the intervention in ways that will trigger its hypothesised mechanisms of action.

As described below, the AMETHIST (Adapted Microplanning to Eliminate Transmission of HIV in Sex Transactions) and SIPP (Safe Inhalation Pipe Provision) studies tested two very different interventions and looked for different health outcomes in diverse populations. Yet the way in which stigmatised, marginalised and often criminalised populations have their agency restricted shares commonalities well-documented in literature, as do the types of interventions known to improve determinants of health and health outcomes for these and similar communities. Harm reduction and peer-led community-level services that work with motivated leaders and their constituencies to reach out to hidden groups, building trust and collaborative relationships, have been shown to be most effective, particularly when participatory and inclusive [30–33].

The AMETHIST trial was a cluster RCT designed to test whether combined microplanning and self-help groups (SHG) could lead to virtual elimination of HIV transmission in sex transactions in Zimbabwe [34]. The underlying rationale used in the AMETHIST logic model was that providing individually tailored outreach services to female sex workers (FSW) based on their levels of risk, proactively supporting their engagement with HIV prevention and treatment services and organising self-help groups to build social support and problem-solving skills would increase FSW’s knowledge, self-efficacy and motivation to engage with clinical care, thus reducing both vulnerability to HIV acquisition and likelihood of onward transmission [35].

The SIPP study [36] is a quasi-experimental structural intervention seeking proof-of-concept for provision of safe inhalation pipes to people who use crack cocaine in the UK. Rationale for the intervention is rooted in principles of harm reduction [37, 38] and community involvement [39, 40], and thus co-designed with users of crack cocaine. The SIPP logic model hypothesises that provision of a safe inhalation kit with pipe, related paraphernalia and information, supported by online training for health providers and peer-to-peer pipe distribution, counselling and referral to drug treatment services would reduce crack injection frequency and harms from shared pipe use, and thus contribute to reduced transmission of blood-borne and respiratory infections, based on causal evidence for these negative health outcomes [41].

Both studies nested prospective process evaluations within their evaluations using the MRC Guidance to collect data on implementation fidelity, feasibility, acceptability and tracked progress across the hypothesised change pathway set out in each logic model. Each used mixed methods, bringing together routine programme statistics, qualitative interviews and field notes, and questions on exposure and perceptions of intervention activities integrated into endline surveys. Both interventions were further likely to be highly sensitive to localised circumstances, particularly how sex work and drug use are organised and conducted, socially perceived, policed and/or disrupted, thus necessitating comprehensive monitoring of contextual factors and their integration into evaluation findings.

## Results

### Development of the Context Tracker for AMETHIST

The “Context Tracker” tool was developed and introduced in 2020 when the COVID-19 pandemic restricted travel and interpersonal contact in Zimbabwe, roughly 1 year into the trial [42]. The AMETHIST study initially planned in-person observation and face-to-face qualitative interviews with study staff and participants to capture events, local conditions and trends in each study site. As with many process evaluations, we had not explicitly defined features of context nor specific indicators. However, when confronted with unavoidable changes to both the intervention’s delivery and our research, we had to make adaptations to both. We also wanted to document variations in how national COVID-19 restrictions were being implemented across study sites. We did not consider the pandemic itself, as a global event, to be a contextual factor except in how restrictions were imposed and enforced differently and effects of this on local communities, particularly how these interacted with delivery of the intervention and engagement by our participants. This caused us to think in a more structured way about relevant domains of context (related to COVID-19 and beyond) and how to find a useful structure for systematically capturing these, particularly through remote forms of data collection.

We developed a tool that was both standardised (i.e. formatted and applied in the same way at all 22 study sites) and flexible enough to allow for unique features in each site to be explored in greater detail. The conceptual rationale was that we could map out evidence-based domains likely to affect both delivery of and participation in intervention components and track these over time, routinely capturing developments in each domain for each site without the need to travel. We also embedded flexibility by allowing for unexpected sources of influence, particularly given the unprecedented experience of a global pandemic and growing evidence of how responses in many settings intersected with existing forms of discrimination, stigma and blame [43, 44].

**Fig. 1:**
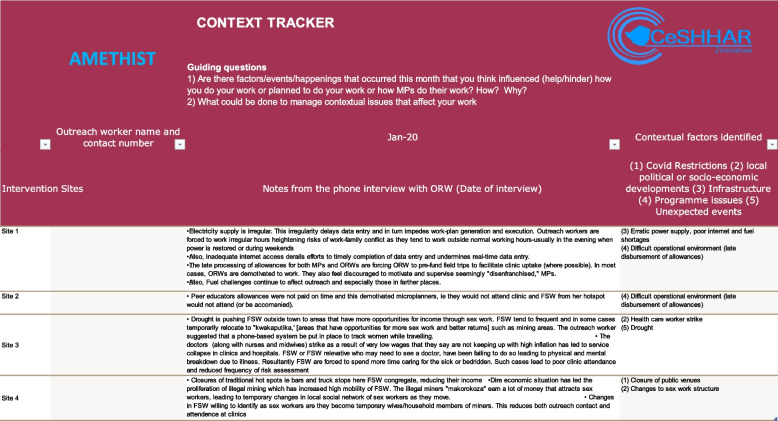
AMETHIST Context Tracker extract

The tool consisted of an Excel database (see Fig. 1) and accompanying interview topic guide that was administered monthly by phone to on-site, purposively selected intervention staff and participants; the spreadsheet was populated during the telephone interview and data extracted, reviewed and synthesised quarterly. The original context domains identified for the study were COVID-related restrictions and their effects; local political or socio-economic developments; infrastructure; institutional/programme issues; and unexpected events. These were selected based on our familiarity with types of differences across the sites and early observations of how COVID-19 interacted with local conditions in ways we predicted would affect delivery and outcomes of the intervention.

The topic guide started with two open-ended questions used to explore each domain: “1) Are there factors/events/happenings that occurred this month that you think influenced (helped/hindered) how you do your work or planned to do your work or how the intervention functioned? How? Why? And 2) What could be done to manage contextual issues that affect the work?” These were followed up every month over 18 months by specific probes to check whether each recorded item was still relevant or had been resolved/changed. Follow-up questions were iteratively developed each month following review of previous entries. By allowing for open-ended responses and longitudinal tracking, the tool could capture the evolving nature of these social dynamics and their impact on the intervention. During the height of the pandemic, however, our topic guide included 8 “standing probes” to capture contextual variations across study sites in socio-economic impacts (changes to sex work), policy (which restrictions were introduced and how enforced), epidemiology (changes to HIV prevention and treatment practices) and accessibility of healthcare (engagement with both intervention and other services); these are listed in Table 1.

**Table 1:** COVID-related questions in Context Tracker

Question	Probes
1. How is business (sex work) going these days?	• Fluctuations in costs and number of clients/week?
	• What is the cost—short/long time, number of clients/weeks?
	• Increasing or not, why?
	• Locations where clients are found/where sex takes place?
2. How is social distancing practised in your business?	• How does that look like for you?
	• How did you practise self-isolation?
3. Do you think your clients are practising social distancing—How does that work in your business?	• Are clients practising social distancing?
	• How does this affect demand for sex?
4. Is the COVID epidemic changing your condom needs these days?	• In what ways and how does it affect how you do business?
5. Have you made any changes to how you provide sex to clients these days?	• What are these?
	• Are you taking any measures to prevent COVID transmission?
6. Have you been to the clinic since COVID epidemic started?	• How was the experience?
	• What differences (if any) were there compared to before?
7. Where else are you getting services? What about other sisters [FSW] in your area?	• Which services are they getting?
	• How?
	• Reasons for change?
8. Are there any stories or experiences of things that have happened as a result of COVID that you would like to share?	[WAIT until respondent is finished before exploring]
	• Violence?
	• Police harassment?
	• Forced removals/deportations?
	• Service disruptions?

Study sites were listed in rows and time periods in columns. Notes were taken during the interview by hand then transferred in bullet-point or summary form into the spreadsheet. Content analysis (both quantitative and qualitative) compared experiences between and within sites, identifying emerging themes for each domain and similarities and differences across time and place. The Context Tracker naturally lends itself to qualitative framework analysis given that it presents key data in “charting” format. The next steps entail mapping and interpreting the organised data to identify patterns, relationships and insights [45]. Figure 1 provides a snapshot from 1 month’s use of the tool, with locations anonymised.

The tool proved useful for integrating data on pre-identified contextual domains with open-ended exploration of emerging issues that might influence the delivery or mechanisms of the intervention. For example, we were able to understand variations in FSW’s access to health care in different sites related to how COVID-19 travel restrictions were locally enforced by linking routine programme data on clinic attendance to reports in the Context Tracker of where public transport had been halted and/or people were apprehended by police when travelling (“COVID restrictions” domain). We also monitored differences in intervention and comparison sites, such as the fact that 40% of intervention clinics experienced regular power cuts compared to only 10% of comparison sites (“infrastructure” domain). We recorded changes in economic activity and how these affected service uptake. In 5 out of 22 trial sites, an increase in illegal mineral and gold mining changed the structure of sex work as a large number of FSW arrived following the influx of new miners but entered into “temporary marriages” rather than selling sex from bars, truck stops or other “hotspots” (“socio-economic changes” domain). This reduced their willingness to attend targeted services that would identify them as sex workers, but increased the likelihood of condomless sex (within more established relationships). It also contributed to higher levels of gender-based violence as competing gangs of illegal miners fought over access to local resources, including sex partners. These developments were followed over subsequent months and we responded by offering a phone-based peer referral system but continued to struggle to reach those FSW whose movements were circumscribed by fear of violence, which finally reduced after a police crackdown on local machete-wielding groups (“unexpected events” domain).

### Conceptual refinement of the tool

Following the successful experience of using the Context Tracker in Zimbabwe in which two authors (FM and JB) were involved, CV and JB started planning the SIPP process evaluation. While defining the SIPP logic model and identifying relevant indicators, utility of the AMETHIST approach to prospectively capturing pre-specified contextual determinants became clear, as did the need for greater conceptualisation and refinement of the tool.

Following discussion and informal review of the literature around measures of context in process evaluations, we first adopted Rogers et al. definition of “context” as “a multi-dimensional construct encompassing micro, meso and macro level determinants that are pre-existing, dynamic and emergent throughout the implementation process” [5]. We subsequently selected two frameworks to guide further development of the data collection tool, Context and Implementation of Complex Interventions (CICI) [9] and Risk Environments [46, 47].

These frameworks both reflect socio-ecological models where individuals are embedded in family and peer groups that reside in communities, which form culturally distinct societies shaped by the laws, policies and economies of the country or region [48, 49]. They categorise social layers of influence into three levels: macro (the highest social level, e.g. national law), meso (related to the interventions’ organisation or community, e.g. social support networks) and micro (attributes of individuals or peer groups, e.g. risk behaviour). The Risk Environments framework is specific to the experience of marginalised populations, who are often “hidden” or excluded [46]. It was originally developed to design harm reduction services to prevent HIV among injecting drug users, but has since contributed to analysis of vulnerability of FSW [47, 50], men who have sex with men [51] and transgender women [52, 53]. The Risk Environments framework identifies “macro” and “micro” levels, but many of its micro-level determinants are primarily community-based (availability of services, social networks and norms, etc.) and thus can be considered “meso” level, but these levels serve as guides rather than rigid categories. The Risk Environments lens considers how individual engagement in “risky” practices despite awareness of their harmful consequences are shaped by social determinants categorised as physical, social, economic and policy. For example, the policy of criminalising drug users results in aggressive police enforcement that can affect both health risks (rushed consumption, sharing of scarce injecting equipment) and reduce provision of and access to harm reduction services [54, 55]. These frameworks resonated with issues we considered salient for FSW services in Zimbabwe and provision of drug consumption equipment in the UK, and also complemented each other through focusing on measuring context in the CICI and understanding (and acting on) determinants of risk among marginalised and hard-to-reach populations in Risk Environments.

We selected 5 domains across all 3 levels (Table 2) and pre-populated each cell with potential shapers of how interventions are delivered and experienced, focusing on interventions for key populations. Any given research study should identify which of these (or others) to include and how to access the relevant data (background information, routine statistics, qualitative methods); accompanying topic guides can be tailored accordingly, relevant respondents selected, and data collection frequency decided. While the context domains and levels are likely to apply to most studies, the evidence-based determinants used to pre-populate each cell will vary.

**Table 2:** Revised Context Tracker template

**Context domains and levels**	**Micro (individual/peer group)**	**Meso (community)**	**Macro (national/cultural)**
**Physical**	• Availability and quality of housing • Location of sex work (street, brothel, entertainment venue) • Proximity to harm-reduction and other services • Disability	• Urban/peri-urban/rural • Availability of public transport • Distribution of illegal activity (drug purchase and consumption and sex work “hot spots”) • Concentration and location of harm reduction, clinical or outreach services	• Climate/seasonality • National infrastructure (e.g. existence of national health system) • National geography/topography
**Social**	• Socio-demographics (race, ethnicity, social class, gender) • Trust in services or authorities • Peer norms regarding drug use/sex work and related risk (alcohol use) • Peer norms/motivation regarding risk reduction/engagement with services	• Social cohesion/support among target population • Communication networks • Willingness to work together for shared benefit/community mobilisation • Availability of community-led organisations	• Levels of stigma against drug use/sex work • Norms related to gender, sex and sex roles • Elections • Political stability, fragility and/or conflict
**Economic**	• Individual economic status • Access to income/alternative livelihoods • Food security • Charging for sex acts (e.g. higher price for non-use of condoms) • Economic dependency (dependent on partner; have children etc.)	• Structure of drug markets and affordability of safe equipment • Competition between FSW for clients • Local tourism/other industries driving sex work • Functionality of services, i.e. clinic stock-outs • Funding from local associations/residents/NGO • Price of drugs/sex	• Political economy • Resource scarcity for health services, e.g. national stock-outs • Donor/funding environment
**Policy/legal**	• Experience of arrest/harassment by police or other authorities • Resistance to/mitigation of policing and enforcement	• Policing practices including police abuse • Cooperation between services and policy/authorities • Availability of harm reduction services • Existence of “victim friendly” units for legal redress	• Laws criminalising or regulating drug use/purchase and sale of sex • Harm reduction regulations • National strategy or targets re: health equity • Structure of health services, e.g. distribution of public and private
**Epidemiological**	• Risk behaviour • Use of prevention • Drug quality at time of consumption	• Background prevalence rate of HIV, HCV, STI etc • Quality of available drug supply	• National prevalence rates • Underlying population health

The Context Tracker is oriented primarily at meso (community) and micro (peer group) levels, as health interventions for marginalised populations often target local settings to create an enabling environment for change [26, 56]. Approaches at this level include increasing supply of resources (condoms, clean injecting equipment), improving interactions between marginalised communities and health authorities (sensitising medical staff, offering outreach and peer-led services) and catalysing community mobilisation to strengthen collective action on shared priorities (supporting peer leadership and organisational development). There is strong evidence for the effectiveness of peer-led and community-based interventions for criminalised populations, mostly on HIV-related outcomes [30, 57–59]. However, many attempts to replicate successful programmes in new settings have been unsuccessful [26, 60], suggesting contextual attributes still need to be better identified, measured and addressed.

As mentioned, the original tool consisted of an Excel spreadsheet with rows for sites, columns for time periods, and an accompanying interview topic guide as a job aid. The formatting can be adjusted, for instance allocating each site a separate Excel tab with rows and columns recording relevant information at micro, meso and macro levels for each domain. Ideally, the Context Tracker should become a living document to open interactive space for interviewer (social scientist) and interviewees to maintain a regular dialogue on contextual factors. Where possible, several respondents should be consulted to triangulate findings (in AMETHIST, these were outreach and peer staff and FSW participants). It should remain both structured (with set domains and interview schedule) and flexible (guided by iterative questions and allowing for addition of domains when required). The spreadsheet has an element of “built in” charting of the data, facilitating framework analysis for comparison between cases (e.g. sites) and identification of trends over time [45, 61]. Emerging findings can also be fed back into the intervention for course-correction and appropriate adaptations made to intervention delivery, as recommended by the original MRC Guidance on process evaluation [2].

### Adaptation and use in SIPP

COVID-19 restrictions were no longer in place by the time of the SIPP study, making it possible to adapt the COVID Tracker for in-person qualitative data collection, including extensive field observation. The research team chose to use a word document rather than Excel spreadsheet for greater ease of inserting long excerpts of written text, with a separate table for each of the 6 study sites (3 intervention and 3 comparison). There was a separate row for each domain, with columns for “micro” and “meso” levels. As all the sites are within the UK, we did not expect national-level (macro) differences. Two further columns were added, “Relevant to intervention (fidelity, feasibility, acceptability)” and “Related to research activities (adherence to study protocol, potential contamination)”. These additional columns allowed for capturing information that seemed directly linked to delivering the intervention or completing the study as planned, for example, where local peer outreach workers appeared to act as “gatekeepers”, limiting the study’s access to some community members, or where there were breakdowns of communication between the research team and implementors that had to be addressed to maintain access to data (see Table 3 for examples). Rather than use an accompanying topic guide, the SIPP research team integrated topics from the Context Tracker into iterative development of other data collection instruments, mostly interviews, focus groups, field notes and extraction from available programme or other local statistics (e.g. local prevalence rate for hepatitis C and other infections among people who use crack).

**Table 3:** Extract from SIPP Context Tracker

**Site 1**				
**Context domains and levels**	**Micro (individual/peer group)**	**Meso (community)**	**Relevant to intervention (fidelity, feasibility, acceptability)**	**Related to research activities (adherence to study protocol, potential contamination)**
**Physical**	• [Service 1] drug treatment services located in a clinical/sanitised setting; lots of CCTV • [Service 2] drug treatment service more hidden away, about 25 min from the city centre. More home-like environment. There is a clinic room for testing, spaces for clients to sit and hang out and offices in the back	• Decent public transport connections into city (tram and bus) • [Service 1] has a van for outreach services including to hostels, home visits and as a mobile needle exchange in grocery store parking lots and other community areas • [Service 1] reaches around 350 crack users. Sees around 100 clients a month for the needle exchange • [Service 2] does street outreach 3 nights a week, delivering food, drinks, condoms and lube to sex workers. Sees about 50–60 clients a month. July/August 23—unable to do outreach due to broken van	• Outreach and fixed site drug services available • (August 23)—[service 2] van broken. Unable to do outreach and rely on drop-in service	• (May 23) two of the three staff doing survey recruitment at [location name] left their position or stepped back from the study
**Social**	• [Service 1] sees mostly white British males between the ages of 20–50. A large number are or have experienced homelessness in their life. A smaller number have been incarcerated at some point • [Service 2]’s population is majority women between 30 and 50		• Risk of peer network acting as “gatekeepers”. Peer network becoming hierarchical • (Apr 23) peer lanyards issued with police referral numbers to ensure they can work without police harassment	• Baseline survey using peer research and drug treatment survey as planned • Difficult interactions between peers and research team at times • (May 23) bank holidays through Apr/May affected [service 2]’s ability to do surveys as they are only open Monday/Thursday
**Economic**	• Sense of good food security. Lots of additional services provided in the city (centre) through hostels/kitchens	• (October 22) problem with equipment supply • HS offer other “services” like tea/coffee, chocolate, sweets, dog food, phone charging, hot drinks, clothes, filling up water	• Services appear adequately resourced	• (Mar 23) study costs not fully understood by peers. Tensions around study reimbursement, e.g. why travel/taxi can be paid for but not other ancillary expenses (e.g. hoodies)
**Policy/legal/health system**	• Little indication of police harassment • (May 23) visit to police to give briefing • Jun 23—largely positive reception from police to briefing	• (April 23) indication of some overzealous police • (May 23) police require ongoing communication from study. Visit and talk given to “proactive policing” teams revealed overwhelming support for initiative. Several officers asked whether they would be able to give out information regarding pipes to people who use crack	• Services more open/progressive than other sites	• [Service 1] focuses on needle exchange but is part of a larger organisation, which also includes several services related to drug and alcohol recovery
**Epidemiological**	• (Apr 23) anecdotal evidence of crack + heroin (speedball) smoking • (Dec 22) anecdotal evidence some people include white rum in washing up process	• Summer/autumn 23—notable drop in heroin quality. May impact injection frequency		• (Apr/May 23) [service 1] reports having difficulty contacting participants to set up interviews. Also reports many no longer consistently smoke crack (not eligible)

The SIPP evaluation is still underway, and the Context Tracker updated on a regular basis. Analysis of the data is conducted in real time but will also be examined in parallel with endline survey data on uptake and engagement with the intervention, and qualitative interview and field note data. To date, some of the findings related to different environmental features likely to interact with the underlying SIPP rationale include the fact that in some sites, sex work is closely associated with crack use (“economic” domain), contributing to a different epidemiological and risk profile among the target participants, and differences in availability of public transport, making travel to the drug services easier and less expensive in some sites compared to others (“physical” domain). Furthermore, levels of social cohesion as expressed through whether people who use crack consider there to be a “sense of community” and trust between peers varied across intervention sites (“social” domain). For example, the death of the director of a local service led to power struggles and disagreements on their whole approach to drug treatment; the effects of this disruption on SIPP implementation at that site was captured in real time through the Context Tracker. Finally, we documented how the intervention itself appeared to influence contextual factors such as policing practice, i.e. we documented how in sites that had positive relationships between implementors and the police, people who use crack were newly able to keep their pipes even when arrested for drug use (“policy/legal/health” system domain). Table 3 provides an extract from the SIPP use of the tool.

## Discussion

“Context” has been highlighted as an important determinant of how an intervention is delivered, perceived, received and responded to, with growing attention to ensuring it is better defined and measured in process evaluations and implementation science [4, 62–65]. Numerous conceptual frameworks now exist, and these can be refined and structured in advance for any specific trial based on existing literature. The Context Tracker is one example of how evidence-based contextual determinants drawn from the harm reduction literature can be used to guide data collection and analysis, in this case, for studies targeting marginalised populations that share barriers and facilitators to health interventions despite their considerable diversity.

While the tool itself may prove useful to others planning process evaluations of complex interventions for “key populations” or other marginalised groups, we consider the broader approach likely to have useful applications for a more general research audience. The Context Tracker demonstrates how to pre-plan integration of context measures into process evaluation using any relevant framework combined with existing evidence and prior experience. While we selected the CICI and Risk Environments frameworks, other models would also be useful. A recent scoping review identified 17 separate frameworks that include contextual determinants [4]. For example, Normalisation Process Theory focuses on factors to consider when moving from testing interventions to embedding them into routine practice [66]. The Consolidated Framework for Implementation Research designates “outer” and “inner” settings [62], while Promoting Action on Research Implementation in Health Services (PARiHS) considers “culture”, “leadership” and “evaluation” as the primary dimensions of the environment into which an intervention is introduced [67]. These and other frameworks offer useful conceptual approaches to researchers, but they remain abstract. Combining them with use of the Context Tracker or an adapted version of it could help translate these frameworks into useful data collection strategies “on the ground”.

At the time that we developed the Context Tracker, we had not read the approach described by Craig et al. [68] that offers a useful categorisation of possible domains. This guideline makes clear that “the features of context that need to be taken into account are intervention specific: not every aspect of context is relevant in every case” (p. 6). It suggests that there is less value in rigid standardisation than early consideration of which features are likely to interact with any given intervention. Familiarity with existing literature and the proposed intervention’s underpinning logic model should enable selection of relevant factors. Despite calls for greater consistency in terminology by others [4, 5], we agree with Craig et al. that the aim should not be to standardise conceptualisation of context, but to encourage early reflection during the design of process evaluation measures for each specific intervention study, thus strengthening the quality of data on context. We hope to expand on the utility of Craig et al.’s guidance by offering greater detail on the mechanics of moving from identification of relevant domains to operationalising them during fieldwork.

A strength of the Context Tracker is that it was initially developed by necessity, when circumstances beyond the control of a research team mandated not just a change of data collection method, but a conceptual re-think for how to capture real-time effects of COVID-19 restrictions on both AMETHIST implementation and research activities. This led to an action-oriented solution rooted in the experience of field work in challenging settings. Because the Context Tracker originated in Zimbabwe, it is responsive to realities of conducting research in resource constrained settings, often marked by poor infrastructure, low internet connectivity and unreliable transport. Potential weaknesses include using the tool too rigidly, without adjusting it for different interventions. It is also possible to get overly bogged down in the categorisation of different shapers as “micro” or “meso”, “socio-economic” or “policy” which could lead to unproductive theoretical debates rather than useful guidance in the field. Finally, while designed to facilitate the process of collecting data and simplify its presentation, the Context Tracker in no way reduces the time and effort required by meaningful data analysis and interpretation and would be misused if considered a “short cut” to considering how interventions are embedded in and interact with dynamic social settings.

We intend the Context Tracker to be relevant in low- and high-income settings and applicable to both qualitative and quantitative analyses. While the data collected can be open-ended text conducive to thematic analysis in smaller-scale and more ethnographic research, its database format allows for prespecifying a limited number of contextual variables for each domain, facilitating quantitative analysis as well as categorisation of contextual attributes useful for innovations in statistical analysis of process evaluation data, e.g. moderated mediation analysis [69]. Although initially introduced to overcome the challenges of fieldwork under COVID-19, the adapted and refined Context Tracker has potential to improve quality and robustness of process evaluations conducted both in more predictable and stable circumstances, but can remain useful should a pandemic or other disruption occur requiring rapid transition to remote data collection.

## Conclusions

While the use of Theory of Change and similar logic models for complex health interventions has helped improve description of interventions and their underlying mechanisms of action, much of the important role of “context” in shaping and explaining what happens in regard to being able to trigger those mechanisms remains opaque in many studies. The accumulation of useful concepts and frameworks can seem overwhelming and abstract. The Context Tracker offers a novel approach to designing and using a tool within process evaluations that provides some structure without removing the potential for iterative analysis and flexibility to follow unexpected findings. The primary implication for future research is availability of a tool for capturing relevant contextual domains in intervention studies in a more rigorous and systematic way, ensuring consistency in measures over time. This will facilitate the difficult task of interpreting the diverse ways that local environments interact with newly introduced programme components, creating facilitators and barriers to implementation, affecting engagement and uptake, and triggering both hypothesised and unexpected mechanisms of action.

## Data Availability

Data sharing is not applicable to this article as no datasets were generated or analysed during the current study.

## Abbreviations

AMETHIST: Adapted Microplanning to Eliminate Transmission of HIV in Sex Transactions
CICI: Context and Implementation of Complex Interventions
FSW: Female sex worker
HIV: Human immunodeficiency virus
MRC: Medical Research Council
PARiHS: Promoting Action on Research Implementation in Health Services
PrEP: Pre-exposure prophylaxis
RCT: Randomised controlled trial
SHG: Self Help Group
SIPP: Safe Inhalation Pipe Provision
